# Plastic and Cosmetic Surgery

**Published:** 1911-05

**Authors:** 


**Plastic and Cosmetic Surgery.** By **Frederick Strange Kolle,** M.D., author of "The x-Rays; their Production and Application," Octavo pp. 532. Illustrated. New York and London: D. Appleton & Co. 1911. (Cloth, $5.00.)

The author has placed before the profession a thoroughly practical and concise treatise on plastic and cosmetic surgery. The importance of this branch of practice is undeniable but heretofore the literature on the subject has been widely scattered. Kolle begins his book of more than 500 pages with an interesting historical resumé, following it with chapters on "Requirements for an Operation," "Wound Dressings," "Surgery of the Eyelids," "Ear," "Nose," "Lips," "Mouth," and "Cheeks." One hundred and thirty pages are given to a discussion of hydrocarbon prothesis and every method of paraffin injection for the correction of facial deformities is noticed. Electrolysis in dermatology with a complete explanation of the methods of procedure and instruments required closes the volume. Here again is one of those particularly valuable and practical works known to reviewers as "worth while books." J. A. R.

